# Artificial Anæsthesia

**Published:** 1878-06

**Authors:** 


*The Advantages and Accidents of Artificial Anæsthesia.* By LAWRENCE TURNBULL, M. D. Philadelphia: Lindsay & Blakiston, 1878. Pp. 200.

This is a manual of Anæsthetic agents and their modes of administration, considering their relative risk, tests of purity, treatment of Asphyxia, Spasm of the glottis, Syncope, and their medico-legal relations. It is well written, shows research, and the conclusions are safe to follow. In reference to the use of chloroform, the author concludes: "I would limit the use of this most potent of all the anæsthetics to very young children. To puerperal eclampsia in very violent convulsions, in male adults, or in females during delivery, where rapidity of dilatation of the os uteri is absolutely necessary to save the mother's life. In some rare cases of painful operation, where, after continued efforts, no complete insensibility can be produced by ether, I would feel justified in the use of a portion of chloroform on a clean sponge or inhaler." He thinks Ether, given with an inhaler is the safest and best anæsthetic and would use no other except in the cases enumerated above.

